# Activation Changes in Zebra Finch (*Taeniopygia guttata*) Brain Areas Evoked by Alterations of the Earth Magnetic Field

**DOI:** 10.1371/journal.pone.0038697

**Published:** 2012-06-05

**Authors:** Nina Keary, Hans-Joachim Bischof

**Affiliations:** Lehrstuhl Verhaltensforschung, Universität Bielefeld, Morgenbreede 45, Bielefeld, Germany; Bowling Green State Universtiy, United States of America

## Abstract

Many animals are able to perceive the earth magnetic field and to use it for orientation and navigation within the environment. The mechanisms underlying the perception and processing of magnetic field information within the brain have been thoroughly studied, especially in birds, but are still obscure. Three hypotheses are currently discussed, dealing with ferromagnetic particles in the beak of birds, with the same sort of particles within the lagena organs, or describing magnetically influenced radical-pair processes within retinal photopigments. Each hypothesis is related to a well-known sensory organ and claims parallel processing of magnetic field information with somatosensory, vestibular and visual input, respectively. Changes in activation within nuclei of the respective sensory systems have been shown previously. Most of these previous experiments employed intensity enhanced magnetic stimuli or lesions. We here exposed unrestrained zebra finches to either a stationary or a rotating magnetic field of the local intensity and inclination. C-Fos was used as an activity marker to examine whether the two treatments led to differences in fourteen brain areas including nuclei of the somatosensory, vestibular and visual system. An ANOVA revealed an overall effect of treatment, indicating that the magnetic field change was perceived by the birds. While the differences were too small to be significant in most areas, a significant enhancement of activation by the rotating stimulus was found in a hippocampal subdivision. Part of the hyperpallium showed a strong, nearly significant, increase. Our results are compatible with previous studies demonstrating an involvement of at least three different sensory systems in earth magnetic field perception and suggest that these systems, probably less elaborated, may also be found in nonmigrating birds.

## Introduction

Wiltschko and Merkel [1] were the first to publish experiments demonstrating the use of the earth magnetic field for spatial orientation. Since then, numerous studies have shown that the magnetic sense is not a specialty of a few species. It can be demonstrated in all vertebrate classes, from fish (e.g. Sockeye Salmons, *Oncorhynchus nerka*; [2]) over amphibians (e.g. European Toads, *Bufo bufo*; [3], [4]) and reptiles (Loggerhead Turtles, *Caretta caretta*; [5]) to birds (e.g. European Robins, *Erithacus rubecula*; [1], [6]) and mammals (e.g. Common Mole Rats, *Fukomys*, formerly known as *Cryptomys hottentotus*; [7]). It was found that earth magnetic field information is used either for compass orientation or for the construction of a navigational map (review: [8]).

While there is no doubt that earth magnetic field information can be detected by animals and is used for orientation in space, the question of how this information is perceived and processed by the central nervous system is not answered as yet. This is mainly due to the lacking knowledge of a special receptor or sensory organ which is stimulated by the earth magnetic field and transforms magnetic into electric signals appropriate for information processing within the central nervous system. Based on research mainly performed on birds, three hypotheses have been put forward, which all rely on convincing experimental evidence. As yet, it is not possible to favor one of the theories over the others. One way out of this dilemma is at present to assume that each of the three perception channels may serve a different task.

One of the first ideas how magnetic information could be transformed into neuronal activity was reported by Presti and Pettigrew [9] who claimed that magnetic particles found in the neck of pigeons could be moved by the forces of the magnetic field, and that these forces might be transformed into neuronal activation by somatosensory nerves. Hanzlik et al. [10] identified small crystals of superparamagnetic magnetite (SPM) in the beak of pigeons (*Columba livia*). Similar results were described by Williams and Wild [11] in zebra finches (*Taeniopygia guttata*). Fleissner et al. [12] revealed details of the arrangement and structure of these crystals located within endings of the ophthalmic branch of the trigeminal nerve in the pigeon. Their findings led them to presume that this perception system contributes to a magnetic “map” sense instead of being part of the magnetic compass system located in the eye, which was mainly described by the Wiltschkos and colleagues (review: [13]). According to Fleissner et al [12], SPM clusters are distorted by natural intensity changes of the geomagnetic field [14]. Since these clusters seem to be attached to the cell membrane by tiny strands of fibers, the distortion could be mediated to mechanosensitive ion channels.

Experiments mainly performed by W. and R. Wiltschko and coworkers (e.g [15], [16]), but also backed by other labs, demonstrated that in accordance with an idea originally put forward by Schulten and Windemuth [17] and later worked out in detail by Ritz et al [18], special pigments within photoreceptors of the retina could serve as receivers of magnetic information. According to this idea, light induced radical pair processes take place within such pigments. The outcome of these radical pair processes is affected by the earth magnetic field and depends upon the angle between the orientation of the photoreceptor and that of the magnetic field lines. According to Ritz et al., parallel processing of visual and magnetic field information in the avian brain leads to a visual percept superimposed by a magnetic percept, possibly manifesting itself through a pattern of brighter and darker shadows on the visual scene, which is related to the orientation of the animal within the magnetic field.

According to the theory, the radical pair process depends on the incidence of light. Indeed, young homing pigeons displaced from the home loft to the releasing site under normal daylight conditions were significantly oriented homewards when released, while pigeons transported in total darkness showed a random distribution of vanishing bearings [19]. By exposing the birds to different monochromatic wavelength, Wiltschko et al. [20] showed a wavelength dependency of the magnetoreception of Silvereyes (*Zosterops lateralis*). This was confirmed in the pigeon [21] and the domestic chicken (*Gallus gallus*; [22]). All these species were disoriented when tested under monochromatic light of lower, but not higher wavelengths. Direct evidence for the involvement of the radical pair process in magnetic field perception was obtained from experiments on European robins [23], chickens [22] and zebra finches [24]. In these experiments, either the natural urge to fly into a particular direction during migrating season was taken advantage of, or the animals were trained to locate a desired object within a test arena by orienting solely on the ambient magnetic field. After having performed the task, a magnetic field oscillating with high frequency was additionally applied. Since oscillating magnetic fields in the MHz range disturb radical pair mechanisms [25] but do not affect magnetic particles, they are well suited to distinguish between the two proposed perception mechanisms. Superimposing a high frequency oscillating magnetic field led to disorientation of the birds. Thus, a radical pair based process underlies magnetoreception in the above mentioned cases. Very likely, the radical pair process takes place at the receptor molecule cryptochrome [26] which has been shown recently to be located in the violet/blue cone receptors of the retina [27].

Findings which were afterwards ignored for some time showed that the lagena of the avian inner ear also contains magnetic particles which could transmit magnetic information [28]. In fact, ablation of the lagena nerves led to a disruption of pigeons' homing ability [29]. Recently, Wu and Dickman [30] reported that there is also physiological evidence for central processing of information from the magnetoreceptor located in the pigeon lagena organs. Using rotating magnetic field vectors as stimuli and c-Fos as a neuroanatomical activity marker, they were able to identify several brain regions activated by magnetic field stimulation and reduced in activity after extirpation of the lagena.

Common to all three hypotheses is the idea that earth magnetic field information is processed by sensory systems which are predominantly processing other sensory input. Magnetic information which is perceived by the visual system has to be processed, at least in the periphery, by the photoreceptors and the retinal network, which mainly processes visual information. Displacement of magnetic particles in the beak is probably transformed to neuronal activity by parts of the trigeminal system, which is mainly a touch sense, and displacements of such particles within the lagena receptor organ have to be registered by the vestibular system, which is involved in monitoring and control of the body position. It remains open where and how in more central stations of the sensory pathways, the magnetic information is separated from the respective “original” or “main” sensory information of the sensory system serving as transporter of the additional magnetic information.

To elucidate this problem, electrophysiological experiments were conducted in the late 80 s and early 90 s in order to find brain areas responding to magnetic field changes. Semm and colleagues recorded activity changes within the accessory optic system, the optic tectum and the trigeminal nerve system in birds [31]–[33], but the data obtained then were not confirmed as yet. By employing immediate early genes (IEGs), like zenk and c-Fos, as neuroanatomical activity markers, several regions were identified that were activated in response to magnetic field changes. Mouritsen et al. [34] described a cluster of pallial visual regions (“Cluster N”) active during nighttime migration restlessness in garden warblers (*Sylvia borin*) and European robins. “Cluster N” receives input from the retina via the visual thalamofugal pathway [35], and lesions of this area in robins eliminate magnetic field orientation [36]. However, “Cluster N” is not activated during the day [34], not even during daytime migration of a day- and night-migrating bird [37] and can also not be identified in other birds, which have been shown to orient after the magnetic field (e.g. zebra finches; [34]). Zapka et al. [37] therefore suggest that the light-dependent magnetic compass requiring an active “Cluster N” may only be used during night-time, while another magnetosensory mechanism may be used during the day.

Heyers et al. [38] provide information about brain areas involved in the processing of the magnetic field information received by the beak SPM particle system. According to their results, the activation within and near the principal (PrV) and spinal tract (SpV) nuclei of the trigeminal brainstem complex, which are known to receive primary input from the trigeminal nerve, was enhanced after magnetic stimulation. Sectioning of the ophthalmic branch of the trigeminal nerve (V1), which possesses endings in and near the above mentioned areas, resulted in a significantly reduced number of activated neurons. Therefore the trigeminal nerve seems to transfer the magnetic information to the brain, and PrV and SpV are associated with the perception mechanisms based on magnetic particles in the beak of birds.

As mentioned above, Wu and Dickman [30] investigated, based on the experiments of Harada [29], which areas within the pigeon brain might be involved in magnetic field processing received by lagena magnetic particles. Using rotating magnetic field vectors as stimuli and c-Fos as a neuroanatomical activity marker, they were able to identify several brain regions probably involved in processing of such magnetic information. These included several regions of the dorsal thalamus, the hippocampal formation, the hyperpallium as well as vestibular nuclei, which were activated by magnetic field stimulation and less active, if the lagena was extirpated.

The overall picture of the distribution of brain areas involved in the processing of magnetic field information, derived from the above mentioned studies, is still far from clear. The results of the studies investigating the “visual channel” for magnetic field perception are as yet not fully satisfactory mainly because “Cluster N” is obviously a speciality of birds migrating at night [35], [36], [38]. How and where eg pigeons or zebra finches might process the magnetic information mediated by the eye is still unknown. The lagena receptor study [30] deploys quite unnatural magnetic stimuli deviating strongly from the normal fluctuations of the geomagnetic field at a given location, and works with head restrained pigeons. Both, Wu and Dickman [30] as well as Heyers et al. [38] used lesions to investigate the influence of the proposed receptors. What was lacking was an experiment demonstrating the effect of the earth magnetic field on the activity of the brain under natural conditions, as it was performed for example by Liedvogel et al. [39] to examine the lateralization of Cluster N activation in a variety of avian species. We therefore decided to conduct an experiment using natural magnetic stimuli and the zebra finch as experimental subject. This nonmigrating songbird has previously been shown to be able to use magnetic field information for orientation in space [40], and has also provided evidence for the participation of the photoreceptor based radical pair process as transducer [24].

In the present experiment, we tested whether horizontal rotation of an otherwise unmodified magnetic field is sufficient to induce activity changes in the brain of unrestrained zebra finches. Controls were birds sitting in an unchanging semi natural magnetic field. Primary candidates for such changes were the areas described by the above mentioned papers. We found out that the applied magnetic conditions were indeed sufficient to induce activity changes in several brain regions of the zebra finch.

## Methods

Fourteen zebra finches of either sex, between six and thirty-two months old, from the institute's stock were used for this study. The birds were housed in individual cages under natural light conditions for up to two weeks prior to the experiments with food and water freely available. All experimental procedures were performed according to the German Law on the Protection of Animals and had been approved by the local government, *Landesamt für Natur, Umwelt und Verbraucherschutz Nordrhein-Westfalen*, approval number AZ 9.93.2.10.36.07.105.

Two to three days before the experiment was conducted the birds were familiarized with the experimental cage by housing them for two to four hours/day singly or in pairs. The experimental cage (18.3×13.6×22.5 cm) was made out of non-magnetic materials only. It comprised a white plastic floor and four walls and a ceiling consisting of wooden bars. The bars had a diameter of 3 mm and an interbar distance of 15 mm. Two perches were positioned inside the cage so that the birds could hop from one perch to another but because of the size of the cage were not able to fly. All integrated screws were also of nonmagnetic materials.

On the day of the experiment a 3-D Helmholtz-coil system (Custom made, Frankfurt a. M. University; Coil control system with DC power supplies, ripple content <0.1%, by Hamann Hard- & Software Development, Plön, Germany) located in one of the university labs was used to generate the magnetic stimuli. There were two parallel coils of 30 windings each with a diameter of 1 m for each dimension. The birds were divided into two groups. One group was subjected to a static normal earth magnetic field (NEMF) set to the local values of Bielefeld, Germany (inclination  = −66°; field intensity  = 42 µT), with the horizontal component shifted from magnetic north by 10° counterclockwise with help of the coil system. The birds of the second group were exposed to a magnetic field with the horizontal component moving stepwise (2000 steps) from 0° to 180° and back within 3 sec (variable earth magnetic field, VEMF), while inclination as well as field intensity remained unchanged. The reason for the slight shift of the horizontal component in the NEMF condition was to anticipate the objection that one of the groups was tested in an active, the other in a passive coil, which could probably have had additional non-controllable effects on the brain activation.

Prior to each trial a high resolution fluxgate sensor (Stefan Mayer Instruments), placed in the center of the Helmholtz coil, was used to calibrate the coil system. Within the center of the system where the cage was placed, the magnetic field variation was below 1%. During the calibration the experimental animal was already sitting inside the experimental cage placed outside of the coil system. After a minimum of 1 h the cage was placed in the center of the coil and the NEMF condition was generated. Animals of both groups remained within this condition for 1 h before the actual test condition was started. For birds of the VEMF group the horizontal component of the field started moving, while nothing changed for the birds of the NEMF group. The test condition continued for 75 min.

About 10 min. after the end of the test the birds were deeply anaesthetized by an intramuscular injection of 0.03 ml Narcoren (Rhone Merioux; 30% in 0.9% sodium chloride). They were perfused transcardially via the left ventricle with phosphate buffered saline (PBS; 0.1 M, pH 7.4, 0.9% sodium chloride) and then with 2% paraformaldehyde (PFA) in PBS, each for 20 min. The head was severed from the body, the skin and eyes were removed, and a small opening was made in the skull, so that the brain could be fixed in 2% PFA in PBS at 4°C overnight. Then the skull was placed in a stereotaxic head holder, specifically designed for zebra finches [41], which was used to provide comparable sectioning planes of the brains and an accurate correspondence to the stereotaxic atlas of the zebra finch brain [42]. The caudal part of the skull was opened and the brain was exposed. After orientation according to the stereotaxic atlas [42], a plane cut (1 mm posterior to the Y-point) was made with a scalpel blade held in an electrode carrier. The brain was then removed from the skull before being post-fixed for several hours in 2% PFA in PBS containing 20% sucrose at 4°C and then transferred to 30% sucrose in PBS overnight. On the following day the right hemisphere was marked by a slight rostocaudal knife cut before the brain was cut into four series of 40 µm thick coronal sections using a freezing microtome.

The sections were collected in PBS and two of the four series were immediately stored in PBS at 4°C as backups while one series was used in a different immunohistochemical procedure. Endogenous peroxidase activity in the sections of the remaining series was blocked by incubation in 0.3% H_2_O_2_ in PBS, followed by several rinses in PBS (5×4 min.). Unspecific binding was blocked with 3% normal goat serum (Vector Labs., S-1000) in PBS for 30 min. After the washing steps in PBS the sections were transferred to a c-Fos-Antibody solution (c-Fos antibody: 1∶2000, Santa Cruz Biotechnology, K-25). The incubation with the primary antibodies was carried out overnight at 4°C on a rotator. This was followed by several washes in PBS, and the second antibody reaction (1∶200, biotinylated anti-rabbit IgG, Vector Labs., BA-1000) in PBS for 1 h at room temperature. After subsequent washes, the signal amplification was performed with the ABC method (Vectastain Elite ABC Kit, Vector Labs., PK 6100). The visualization of the antibody-complex was performed with VIP (Vector, “Very Intense Purple” substrate kit for peroxidase, Vector Labs., SK-4600). This produced a deep purple reaction product confined to the cell nuclei of activated neurons. The free-floating sections were mounted serially on gelatin coated slides and air-dried at 50°C on a heating plate. Thereafter, they were slightly counterstained with methyl-green (Vector methyl-green nuclear counterstain, Vector Labs., H-3402), dehydrated and cover-slipped with DePeX (Serva). Brain sections were examined under a Zeiss Axioscope at final magnification of 100 or 200x. Photomicrographs were taken with a mounted digital camera (Olympus, E 520).

The qualitative evaluation of the distribution of IEG expressing neurons in the following brain areas was undertaken by using a detailed atlas of the zebra finch brain [39]. Hyperpallium apicale (HA), Hyperpallium densocellulare (HD), Hippocampal formation and Parahippocampal area (HP), Nucleus of the basal optic root (nBOR), layers two to four of the Optic tectum (TO), Nucleus sensorius principalis nervi trigemini (PrV). Quantitative analysis was performed after qualitative evaluation.

For HA and HD twelve serial sections (with an equal distance of 120 µm to each other) were selected, six sections from the rostral hyperpallium (5.67–4.83 mm anterior to the Y-Point) and six sections from the subsequent caudal part (4.85–4.05 mm anterior to the Y-Point). Of the ten sections selected for the evaluation of HP five were taken from the rostral hippocampus (3.42–2.74 mm anterior to the Y-Point) and five from a more caudal part (1.99–1.35 mm anterior to the Y-Point). The IEG expressing neurons within nBOR were counted in two sections from 2.25–2.05 mm anterior to the Y-Point. For the evaluation of layers two to four of TO six sections were chosen lying between 2.43–1.59 mm anterior to the Y-Point. PrV could only be identified in ten of the twelve zebra finch brains. Immunoreactive neurons could be counted in four of the VEMF group birds and six of the NEMF group birds. One or two sections were taken for evaluation (0.56–0.36 anterior to the Y-Point).

**Figure 1:**
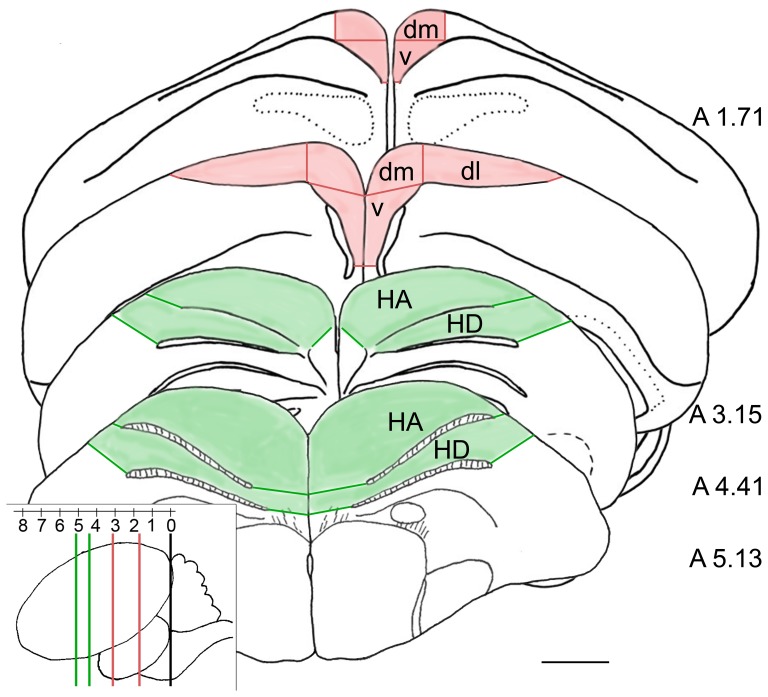
Hyperpallial and Hippocampal subdivisions. Four sections taken from the zebra finch brain atlas, with their coordinates anterior to the Y-Point indicated in mm on the right, depict the middle of the areas defined as rostral and caudal Hyperpallium (A 5.13 and A 4.41) and rostral and caudal Hippocampus (A 3.15 and A 1.71). The extent of HA and HD are shown by the light green background, whereas the confines of the Hippocampal region can be distinguished by the light pink background. The borders of hippocampal subdivisions are indicated by red lines. In the lower left corner the sectional planes of the above displayed sections can be distinguished on the lateral view of a whole zebra finch brain. The vertical black line indicates the Y-Point at 0 mm. The vertical green and red lines indicate the sectional planes of the rostral and caudal Hyperpallium, and the rostral and caudal Hippocampus, respectively. HA  =  Hyperpallium apicale, HD  =  Hyperpallium densocellulare, dm  =  dorsomedial, dl  =  dorsolateral, v  =  ventral. Scale bar  = 1 mm.

Measurements were performed with an ocular grid, the areas defined as detailed below. Since there have been recent reports of only the dorsal part of the hippocampus mainly being involved in the recall of a spatial memory task [43] we decided to subdivide the hippocampal region into a ventral, a dorsomedial and a dorsolateral part for counting of immunoreactive neurons (see figure 1), thus following the basic subdivision promoted by Kahn et al. [44] and Atoji and Wild [45]. In this way we also comprise the parahippocampal area, which arises from the dorsal part of the hippocampal formation and extends laterally, without having to define a clear confine between the two. The subdivisions were defined after assessing the shape of each section. The boundary line between ventral and dorsomedial area extended horizontally from the dorsal end of the ventricle (running alongside the lateral part of the ventral HP and giving it a V-shaped form) to the medial limit of HP where the curvature of the dorsomedial tissue graded into a straight line. In the same way the border between the dorsomedial and lateral HP were defined, stretching roughly vertically from the dorsal end of the ventricle to where the curvature of the dorsomedial tissue became straight and continued laterally. In addition the cell structure in this area changed slightly from larger to smaller size, which was taken as guidance in placing the boundary line. The more caudal part of the HP was divided into a ventral and a dorsomedial part only, as the dorsolateral area was beginning to undergo changes in shape which prevented a unitary placement of the ocular grid used for evaluation. The optic tectum (TO) was also subdivided into three parts for the evaluation, a dorsal, a lateral and a ventral part.

Estimation of cell densities was done by placing an ocular grid onto the different brain areas in such a way that it covered most of their surface. A magnification of 100x was used so that each square covered an area of 0.01 mm^2^. If it was not possible to count within the entire area of one of the small squares, this square was omitted from further calculation. For the optic tectum, a spherical structure of up to thirteen different layers (depending on different definitions by different authors), a different approach had to be taken. Since layers two to four are the initial recipients of incoming information the evaluation was done within these layers. The grid was either 2×10 or 1×10 squares in size depending on the varying width of the layers. It was positioned at a dorsal and a ventral site, carefully paying attention not to place it too far medially, since the layers drift apart at their endings. The lateral position included the lateral most curvature of layers two to four placed into the center of the grid. During evaluation the straight grid placed over the curved course of the layers was corrected for by estimating a curved course of the grid by eyesight. As TO neurons are quite small, a magnification of 200x was employed for counting in this region, so that each square of the ocular grid covered an area of 0.0025 mm^2^.

Several regions were counted a second time with a different counting method in order to make sure the previous method, with large areas covered, did not diminish the outcome of the evaluation. For the second round the rostral HA, rostral HD, caudal HD and the dorsomedial part of the rostral HP were evaluated with a grid of only 2×4 squares in size. This grid was positioned over the estimated highest density of immunoreactive neurons within the region. In order to find out whether an increased sample size would sharpen the contours of the results, two more birds, one belonging to each treatment group, were processed and evaluated with the second counting method. This evaluation was repeated by an independent second researcher to rule out any counting bias of the main interpreter.

After counting, cell densities were standardized to an area of 1 mm^2^. Then, for each brain area evaluated a mean value from all consecutive sections was calculated (fewer sections were used if the brain area in question was damaged or was otherwise affected by the free-floating immunohistochemical procedures or while mounting on slides). Such means were calculated for both hemispheres separately. The individual bird means of each hemisphere were considered as overall ratings for the number of IEG-immunoreactive neurons in these areas and were taken for further statistical analysis.

For statistical analysis, a multifactorial ANOVA was performed with Statistica 6.1 (StatSoftInc, Tulsa, U.S.A.), with Treatment, Area, and Hemisphere as factors. Posthoc LSD (Fisher's Least Significance Difference) tests on the treatment differences within the different areas were performed with the same program.

## Results

**Figure 2:**
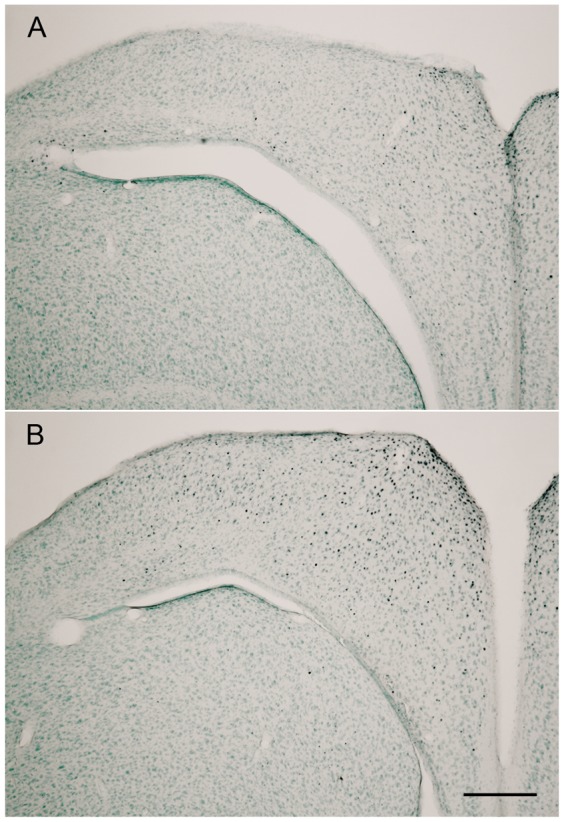
C-fos IR-neurons within the dorsomedial rostral Hippocampus. A. Only scattered activation is found in the brain of a NEMF group bird. B. Far more dark stained cell nuclei can be seen in the brain of the VEMF group bird. Scale bar  = 300 µm, applies to A and B.

The expression of the immediate early gene product c-Fos was visualized in the brain of fourteen birds. The c-Fos containing cell nuclei were stained deep purple and were well distinguishable against the light methyl green background staining in all cases (see figure 2). Our control counts, as described above, showed that neither the counting of smaller areas within four of the brain regions investigated, nor the evaluation performed by a second person revealed differences to the results of the main counting procedure. We therefore describe only the results of the latter one.

**Figure 3:**
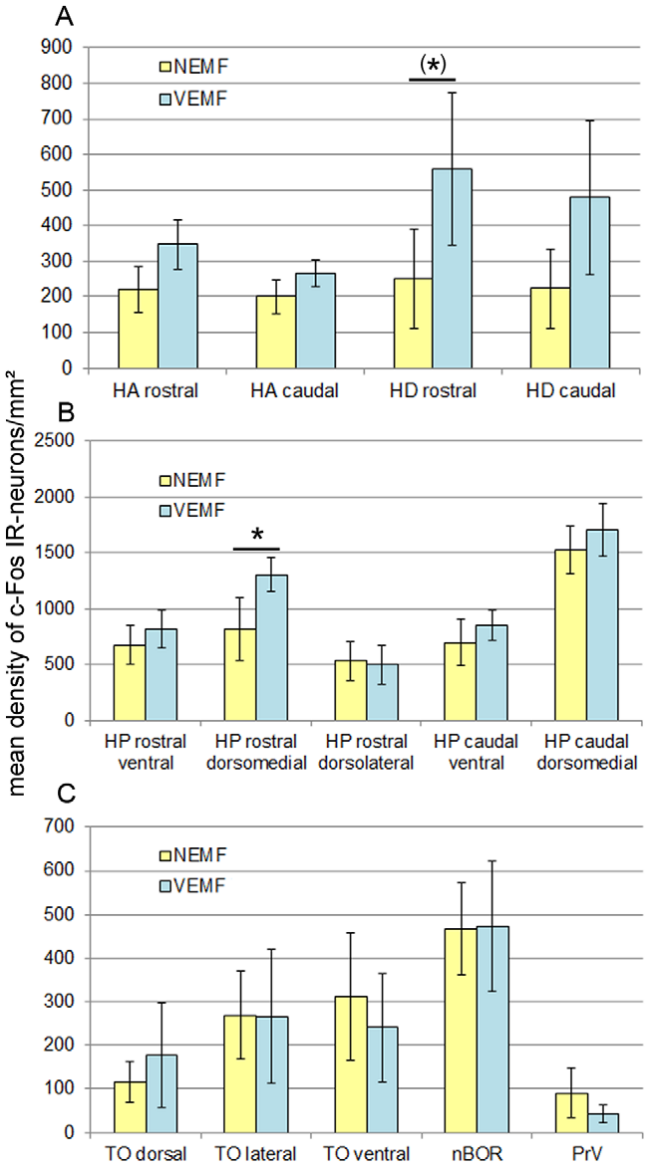
Quantification of c-Fos IR-neurons in two groups of zebra finches. Differences in the density of c-fos-activated neurons within 14 brain regions between two groups of birds, exposed to a stationary normal earth magnetic field (NEMF) or a variable earth magnetic field (VEMF) are shown. The density of c-Fos IR-neurons is given as a mean/mm^2^ ± SEM. A. C-Fos activated neurons in the rostral and caudal parts of Hyperpallium apicale and Hyperpallium densocellulare. All four regions show an increased density in birds of the VEMF group with an almost significant difference within the rostral HD. B. C-Fos activated neurons in the ventral, dorsomedial and dorsolateral part of the rostral Hippocampal formation and the ventral and dorsomedial part of the caudal Hippocampal formation. Of the five regions four show an increased density in birds of the VEMF group with a significant difference within the dorsomedial part of rostral hippocampus. A slight decrease can be seen within the dorsolateral rostral hippocampus. C. C-Fos activated neurons in layers two to four of the dorsal, lateral and ventral Optic tectum, the nucleus of the basal optic root and the principal nucleus of the trigeminal brainstem complex. The dorsal TO shows an increased number in c-Fos positive cells in the VEMF group birds. Equal numbers of activated neurons are found in the lateral TO and the nBOR, whereas a decreased density can be seen in the ventral TO and PrV.

The ANOVA revealed an overall effect of treatment (F (1,11) = 7.269, p = 0.007). This clearly indicates that the variable magnetic field in the VEMF condition did get noticed by the experimental birds. There also was a significant effect of area (F (13,11) = 1.089, p = 0.001), which is not surprising since each area gets activated to a very different degree as can be seen from figure 3. Activation of the two hemispheres was not significantly different from one another (F (1,11) = 1.08, p = 0.298) and none of the interactions of the three factors yielded significant differences. Posthoc LSD Tests were conducted to find out if the density of immunoreactive (IR) neurons between the treatment groups was significantly different within the areas. This was true for the dorsomedial part of the rostral hippocampus which was significantly more activated in the rotating magnetic field condition (df  = 276, p = 0.003). The rostral Hyperpallium densocellulare showed an almost significant enhancement in the same situation (df  = 276, p = 0.056). In all other areas, differences between the groups were far from significant. The evaluation revealed a slight increase of cell density in nine of the fourteen assessed brain regions of the birds that had been exposed to the variable magnetic field as opposed to those that had been kept in the normal earth magnetic field condition. In two regions the cell density of the two groups was equal and in three regions cell density was lower in the VEMF group birds in comparison with the NEMF group birds. For all areas both hemispheres were counted, but since there were no significant differences between cell densities of the left and the right side, they were taken together in the figure.

## Discussion

Our study was designed to investigate whether a relatively subtle and naturally occurring alteration of the earth magnetic field, a rotation without any change in inclination and intensity, can induce changes of the activation within brain areas of unrestricted, freely moving zebra finches. Due to the experimental design, the variability of the obtained data was quite high. However, our results demonstrate that there is a significant overall effect of the rotation on the activity of the investigated areas, if compared with the brains of control birds which were kept in a nonmoving magnetic field with only a small change in the direction of the magnetic field lines. Most areas showed slight increases in the rotating field condition, a decrease or no change was seen only in a few (fig. 3). The observed changes are most probably not due to differences in activity [46] because in another experiment under identical conditions the number of hops and head turns did not differ between the groups (unpublished results). Posthoc tests demonstrated that in most areas the effects were too weak to be significant, but there were two areas showing significant or almost significant enhancements in the number of c-Fos expressing neurons, an indicator for an activity enhancement of the investigated area. One of these areas was the dorsomedial rostral hippocampus, the other the rostral part of the Hyperpallium densocellulare.

The avian hippocampus has been shown to be involved in spatial orientation in numerous experiments, for example in pigeon navigation (e.g [47], [48]) or in food storing birds (e.g [49], [50]). In the zebra finch, hippocampal lesions severely affect spatial learning [51] and the zebra finch hippocampus is strongly activated, as shown by c-Fos experiments, during acquisition and retention of spatial tasks [52]. Given these results in combination with the fact that zebra finches can use the earth magnetic field for spatial orientation [40] it was not very surprising that there was a significant enhancement of hippocampal c-Fos expression by experimental rotation of the earth magnetic field. This finding is consistent with a recent finding by Vargas et al. [53] who detected single units within the hippocampal formation of pigeons reacting to direction changes of the earth magnetic field. Although they described only a few reactive neurons, these were placed in the hippocampal region where we also found the highest activation changes (see fig. 2 and fig. 1 of [53]). Concerning location, our activity patterns were similar but fainter compared with those shown by Wu and Dickman [30] who also described an effect of the earth magnetic field on c-Fos activation within the avian hippocampus. According to their results, hippocampal activity changes were dependent on the proper functioning of the inner ear gravity receptors, which contain SPM particles. The inner ear system appeared to be the only source for the magnetic information transferred to hippocampus because a lesion of this system reduced the hippocampal activation in pigeons to rest levels. The difference in the hippocampal activity levels between the present study and that of Wu and Dickman [30] is probably due to the different strength of the magnetic field applied.

Another brain region, the Hyperpallium densocellulare, showed an almost significant enhancement of c-Fos activation when the birds were sitting in a rotating magnetic field. A part of the hyperpallium, the so called “Cluster N”, has recently been proposed as a part of the avian earth magnetic field orientation system. “Cluster N” is a part of the thalamofugal visual system [35]. Although it was not clear for some time whether the area was indeed involved in processing of magnetic field information [34], a recent experiment clearly indicated such an involvement [37] at least in night-time migration. Whether the activation enhancement in our study is identical in location with “Cluster N” cannot definitely be concluded from our data. However, the location of the observed enhancement corresponds to that identified as visual projection area by our evoked potential studies investigating the hyperpallium [54]. This suggests that the thalamofugal system is, comparable to night migrating songbirds, the transporter for magnetic field information also in zebra finches.

One of the nuclei of the trigeminal system, the PrV, which receives input from the beak magnetic field perception system, has recently been shown [38] to express enhanced IEG activation when birds were exposed to a permanently changing magnetic field if compared to a zero field, a condition where the earth magnetic field is fully eliminated. In contrast, there was no enhancement found in our study. This could be due to the fact that there was an intensity change made in the Heyers et al. [38] experiment, while the intensity remained the same in both conditions of our experiment. According to Semm and Beason [33] and Wiltschko et al. [55], the trigeminal system may especially be tuned to measure magnetic field intensities (but see Falkenberg et al. [56]). Also, the difference between the stimulation by a zero magnetic field and a moving field may be bigger than that between a static and a moving one. Thus, our stimulus change might have in some cases been too small to induce a measurable difference.

The visual system based magnetic perception system as well as that originating at the lagena receptors are both most probably measuring directional parameters of the magnetic field, either the inclination or the direction of the magnetic field lines, or both. Because we changed one of the parameters, the direction, in our study, an effect on the hippocampus and the hyperpallium was to be expected.

Our results are thus consistent with those obtained in the other studies dealing with the different sensory systems carrying piggyback information on the properties of the earth magnetic field. They suggest that under normal conditions, all systems are working in parallel, two systems involved in determination of directional properties of the magnetic field, the third probably dealing with its intensity. As yet, it is not known whether there is a central structure collecting all this information. Because hippocampus is known to be involved in almost every aspect of spatial orientation [57], it may well be that it is also integrating earth magnetic field information into a multimodal spatial map. This may be supported by our own experiments on spatial memory in zebra finches indicating that there is a strong interaction between hyperpallium and the hippocampus; lesions to one or the other of these two structures have the same deleterious effect on both, acquisition and retention of spatial memory [58]. Information carried by the visual system may therefore be transported to hippocampus by this link. Information from the lagena system or the trigeminal system reaches hippocampus in birds via thalamic relay nuclei [30], thus, an integrative function of this structure seems plausible.

It might be, however, not necessary to postulate a common terminal for the different streams of magnetic information. Instead, the concomitant activation of brain areas at the same time could be sufficient to indicate the relatedness of the information from different channels. This idea, the so called binding theory, has mainly been put forward by Singer and colleagues (e.g [59]–[62]). According to their findings, complex stimuli elicit oscillatory activation of clusters of neurons simultaneously within different areas of the cortex. By this synchrony, the brain identifies the activity of different brain areas as to belong to a single object, and binds them to one internal representation. It is immediately obvious that such a system might be a way to solve the question of how the magnetic information could be separated from the original information of the carrier system [63], and it may also help to attenuate the relatively small signals above noise level.

Taken together, our results add to the evidence that zebra finches as nonmigrating songbirds are able to use the magnetic field for orientation, and they are giving the first hints where in the brain of these birds earth magnetic field information is processed. We do not find as strong effects as have been shown in migrating birds; this may have been due to methodological differences, but could also indicate that the involved systems are not so much elaborated in the non-specialist. That the systems are existing in nonmigrating songbirds, however, supports the idea of a very early evolution of the avian magnetic compass and the underlying neuronal system, probably already 95 million years ago in the late cretaceous [16], [64].
